# *Panax notoginseng* Root Cell Death Caused by the Autotoxic Ginsenoside Rg_1_ Is Due to Over-Accumulation of ROS, as Revealed by Transcriptomic and Cellular Approaches

**DOI:** 10.3389/fpls.2018.00264

**Published:** 2018-02-28

**Authors:** Min Yang, Youcong Chuan, Cunwu Guo, Jingjing Liao, Yanguo Xu, Xinyue Mei, Yixiang Liu, Huichuan Huang, Xiahong He, Shusheng Zhu

**Affiliations:** ^1^State Key Laboratory for Conservation and Utilization of Bio-Resources in Yunnan, Yunnan Agricultural University, Kunming, China; ^2^Key Laboratory for Agro-biodiversity and Pest Control of the Ministry of Education, Yunnan Agricultural University, Kunming, China

**Keywords:** antioxidant, reactive oxygen species, autotoxicity, ginsenosides, cell wall, transcriptomics, *Panax notoginseng*

## Abstract

*Panax notoginseng* is a highly valuable medicinal herb, but its culture is strongly hindered by replant failure, mainly due to autotoxicity. Deciphering the response mechanisms of plants to autotoxins is critical for overcoming the observed autotoxicity. Here, we elucidated the response of *P. notoginseng* to the autotoxic ginsenoside Rg_1_ via transcriptomic and cellular approaches. Cellular analyses demonstrated that Rg_1_ inhibited root growth by disrupting the cell membrane and wall. Transcriptomic analyses confirmed that genes related to the cell membrane, cell wall decomposition and reactive oxygen species (ROS) metabolism were up-regulated by Rg_1_ stress. Further cellular analyses revealed that Rg_1_ induced ROS (${\text{O}}_{2\cdot }^{-}$ and H_2_O_2_) accumulation in root cells by suppressing ascorbate peroxidase (APX) and the activities of enzymes involved in the ascorbate-glutathione (ASC-GSH) cycle. Exogenous antioxidants (ASC and gentiobiose) helped cells scavenge over-accumulated ROS by promoting superoxide dismutase (SOD) activity and the ASC-GSH cycle. Collectively, the autotoxin Rg_1_ caused root cell death by inducing the over-accumulation of ROS, and the use of exogenous antioxidants could represent a strategy for overcoming autotoxicity.

## Introduction

Autotoxicity occurs when a plant releases toxic substances into the environment to inhibit the growth of conspecific plants (Singh et al., 1999) and plays important roles in the regulation of plant biodiversity and productivity in natural systems (Chou, 1999; Singh et al., 1999; Inderjit and Duke, 2003). However, increasing lines of evidence show that crop replanting failure and decreases in yields are associated with autotoxins (Singh et al., 1999). Hundreds of allelochemicals released by plants have been identified as potential autotoxins (Huang et al., 2013). Although the allelopathic mechanisms of some allelochemicals, including single or multiple effects involving oxidative damage, phytohormone activity, DNA damage, photosynthetic and mitochondrial function, or water balance, among other processes (Bais et al., 2003; Weir et al., 2004; Yang et al., 2011), have been elucidated, only a few autotoxins have been studied to determine their mechanism of autotoxicity in plants (Chi et al., 2013; Wu et al., 2015).

Sanqi ginseng (*Panax notoginseng*) is a highly valuable medicinal herb due to its ability to ameliorate blood stasis and improve blood circulation (Wang et al., 2016). To match the increasing level of *P. notoginseng* consumption, large-scale gardens of this species have been established in China in the last three decades. However, Sanqi ginseng plants are strongly hindered by replant failure when new seedlings are established in the fields under consecutive cropping conditions (Yang et al., 2015). Even long replanting intervals of 15–30 years cannot completely eliminate replant failure (Yang et al., 2015). Obstacles to replanting are also prevalent among other *Panax* species, including *P. ginseng* (Asian ginseng), *P. quinquefolium* (American ginseng), *P. japonicas* (Japanese ginseng), and *P. vietnamensis* (Vietnamese ginseng) (Nicol et al., 2003; Bi et al., 2010; Ying et al., 2012). The deterioration of soil physicochemical properties, imbalances of available nutrients, build-up of specific pathogens, and accumulation of autotoxins have been recognized as the factors contributing to this problem (Wu et al., 2008). However, our recent data demonstrated that autotoxicity in land where *P. notoginseng* is continuously cropped, which harms root cells, reduces disease resistance, and eventually affects the yield and quality of the plants, is the main factor inducing replant failure (Yang et al., 2015). Activated charcoal adsorption and microbial degradation of autotoxins have been reported as useful mitigation strategies against autotoxicity in *Panax* species (Li et al., 2014; Yang et al., 2015).

Ginsenosides, a group of secondary metabolites, are triterpenoid saponins found nearly exclusively in *Panax* species (family Araliaceae), including *P. notoginseng, P. ginseng, P. quinquefolium, P. japonicas*, and *P. vietnamensis* (Fujioka et al., 1989; Court et al., 1996; Tran et al., 2001; Wei et al., 2007; Zhou et al., 2007; Christensen, 2009; Qi et al., 2011). Ginsenosides have been reported to serve as the autotoxins responsible for the replanting failure of Sanqi, Asian, and American ginseng (Nicol et al., 2002, 2003; Zhang and Cheng, 2006; Yang et al., 2015). Rg_1_ is one of the most abundant ginsenosides in the rhizosphere soil and root exudates of *P. notoginseng* and shows strong autotoxicity by inhibiting seedling emergence and growth (Yang et al., 2015). However, little is known regarding the response mechanism of root cells to autotoxic ginsenosides, which has hampered the development of effective techniques for overcoming the autotoxicity responsible for the replanting failure of *Panax* plants under sole-cropping systems.

With the development of gene sequencing technology, transcriptomics has become a powerful tool for obtaining large-scale snapshots of transcripts and has been widely applied to plant research (Mochida and Shinozaki, 2012). This approach has many advantages in elucidating the responses of organisms to abiotic pressures (Hirai et al., 2004). In this study, we integrated transcriptomic and cellular approaches to elucidate the responses of root cells of *P. notoginseng* to the autotoxic ginsenoside Rg_1_, which might contribute to the development of new techniques for overcoming the problem of autotoxicity in the field.

## Materials and methods

The field experiment was carried out in Experimental Station of Yunnan Agricultural University. These field studies were authorized by Yunnan Agricultural University, Yunnan, China. No specific permissions were required in these fields. We confirmed that the field experiment and plant materials did not involve endangered or protected species.

### Plant growth

Seeds of *P. notoginseng* were collected from mature plants, sown in the wells of seedling-raising plates, and then incubated in a greenhouse under controlled conditions (20–30°C, day length of 12 h). The soil in the seedling-raising plates consisted of a mixture of six parts field soil, two parts perlite and two parts sand (pH: 6.53; electrical conductivity: 280 μS cm^−1^; 1.6% organic matter; nutrient contents: 105.62 mg kg^−1^ available N, 195.10 mg kg^−1^ available P and 124.24 mg kg^−1^ available K). The plants were initially irrigated once with 50% Hoagland's solution (Hoagland and Arnon, 1950) and watered twice a week with fresh water over the course of the experiment. After 5 months of growth, 8–10 cm-high seedlings were used for subsequent experiments.

### Determination of the Rg_1_ concentration in cultivated soil and its effect on growth and conductivity

Soil samples were collected in October 2014 from a Sanqi ginseng field in Yanshan County (104.33°E, 23.54°N). Ten bulk soil samples were collected from fields in which *P. notoginseng* was consecutively cultivated for 1, 2, or 3 years. Rg_1_ was extracted from the soil with MeOH:H_2_O (80:20) and quantified via HPLC-ESI-MS as described by Yang et al. (2015). The effect of the autotoxic compound Rg_1_ on the biomass of *P. notoginseng* was investigated according to its concentration in cultivated soil. Briefly, the ginsenoside Rg_1_ (purity ≥98%, Guizhou Dida Biological Technology Co.) was dissolved in methanol and then diluted in distilled water to final concentrations of 0.1, 0.5, 1.0, and 5.0 mg L^−1^. Distilled water containing the same concentration of methanol (1.0%) was used as a control. Seedlings of *P. notoginseng* were carefully collected from the soil and washed three times with sterile water. A 100-mL aliquot of Rg_1_ solution was added to each sterile glass bottle (250 mL). Ten seedlings were placed in each bottle, and each treatment included six replicates. The seedlings were incubated in a programmable illuminated incubator under a light/dark (L/D) cycle of 12 h/12 h and a temperature cycle of 25°C/20°C. The conductivity of the hydroponic seedling solution was measured using a conductivity meter (FE30, Mettler Toledo Int., Inc., Switzerland) at 12-h intervals. After incubation for 96 h, the wilt ratio of seedlings was calculated as wilt ratio (%) = 100 × (wilted seedlings/total seedlings), and the reduction in biomass was calculated as reduced biomass = fresh weight before treatment – fresh weight after treatment.

### Vigor staining of root cells

Changes in the vigor of the root cells were monitored using a modified capillary root model, as described by Yang et al. (2015). Briefly, the tip of each fibrous root was incubated with Rg_1_ solution (1.0 mg L^−1^) or sterilized water containing 1.0% methanol at room temperature (24°C). The effect of Rg_1_ on root cell vigor was observed by staining with fluorescein diacetate (FDA) (5 mg L^−1^) and the red fluorescent dye propidium iodide (PI) (5 mg L^−1^) as described in a previous report (Fan et al., 2013). Staining was visualized using a Leica SP5 confocal laser-scanning microscope (excitation at 488 nm and emission above 630 nm; Leica, Wetzlar, Germany) after incubation for 0.5, 1.0, 2.0, or 3.0 h. The experiment was repeated three times, and each replicate included 10 roots.

### Effect of Rg_1_ on the root ultrastructure

Seedling roots were treated with Rg_1_ solution at a concentration of 1.0 mg L^−1^ or with sterilized water containing 1.0% methanol. After incubation for 0, 0.5, 1.0, 3.0, 4.0, 6.0, 12.0, or 24.0 h, the root tips were excised with a sterile razor blade and prepared for viewing under a transmission electron microscope (TEM) as detailed by Zhu et al. (2007). The experiment was repeated three times, and each replicate included 10 roots.

### RNA sequencing and data analysis

Seedling roots were treated with Rg_1_ (1.0 mg L^−1^) or sterilized water containing 1.0% methanol for 0, 3, 12, 24, or 48 h. The fibrous roots were then rapidly harvested from the seedlings at each time point, immediately frozen in liquid nitrogen, and stored at −80°C until RNA extraction. Each treatment included three independent replicates. Total RNA was extracted from fibrous roots using the TRIzol method. Because reference genome was unavailable for *P. notoginseng*, a transcriptome assembly library was constructed as a reference library by mixing equal amounts of RNA from the above 15 samples. The libraries were sequenced on the Illumina HiSeq™ 2000 platform by Gene Denovo Co. (Guangzhou, China). Clean reads were assembled de novo using the Trinity programme (version: r20140413p1). Unigenes were then annotated employing the NCBI NR, NT, Swiss-Prot protein, KEGG, GO and COG databases.

A gene expression analysis was performed in two sequential steps. First, all the clean reads were mapped to the assembled sequences using Bowtie 2–2.2.3 to calculate the read counts for each transcript (Langmead and Salzberg, 2012). The transcript abundance for each gene was then measured and normalized as fragments per kilobase of exon per million fragments mapped (FPKM) values (Mortazavi et al., 2008). The differentially expressed genes (DEGs) between 0 and 3 h, 0 and 12 h, 0 and 24 h, and 0 and 48 h of Rg_1_ treatment were restricted based on a false discovery rate (FDR) ≤ 0.05 and an absolute value of the log_2_Ratio ≥ 2. A gene expression cluster analysis of DEGs was performed using the STEM algorithms (Ernst and Bar-Joseph, 2006). Hierarchical clustering of the DEGs related to the response to ROS, ROS-scavenging enzymes, cell wall decomposition, the cell membrane, xenobiotic detoxification, and protein kinases was performed using the FPKM expression values with the pheatmap programme in the R programme environment (version 2.9.0). The transcriptome datasets can be retrieved from the NCBI SRA database under Project ID PRJNA338825.

### Effects of Rg_1_ and exogenous antioxidants on root growth, oxygen damage, and enzymatic activities

To test the effects of Rg_1_ and exogenous antioxidants [ascorbate (ASC) and gentiobiose] on growth, glass bottles (250 mL) containing 100 g of coarse silica sand (sterilized at 160°C for 3 h) were supplemented with Rg_1_ alone (1.0 mg L^−1^), ASC alone (0, 0.1, 1.0, 10.0, or 50.0 mg L^−1^), gentiobiose alone (0, 0.1, 1.0, 10.0, or 50.0 mg L^−1^), or a combination of Rg_1_ and antioxidants. The seeds were surface-sterilized with 1% sodium hypochlorite for 5 min and washed three times with sterile water. Ten seeds were sown in the silica sand in each bottle, and 10 mL of the respective treatment solution was added. All the treatments were incubated in a programmable illuminated incubator as describe above. After incubation for 30 days, the germination rate and plant biomass were measured. The experiments were performed three times with six replicates. The resultant data were analyzed via one-way analysis of variance followed by *post-hoc* Duncan's test (*p* < 0.05).

To test the effect of Rg_1_ and exogenous antioxidants in relation to oxygen damage and enzymatic activities, seedling roots were exposed to Rg_1_ (1.0 mg L^−1^), either alone or with ASC (1.0 mg L^−1^) or gentiobiose (10.0 mg L^−1^). After incubation at room temperature (24°C) for 0, 3, 12, 24, or 48 h, the accumulation of ROS (superoxide and H_2_O_2_) in the roots was observed. Superoxide was stained with 0.5 mg mL^−1^ nitrotetrazolium blue chloride (NBT) in 10 mM potassium phosphate buffer (pH 7.8), as described by Dutilleul et al. (2003), and then observed with a compound microscope (Leica DM2000, Wetzlar, Germany). The H_2_O_2_ content was measured spectrophotometrically at 410 nm according to the procedure described by Shi et al. (2015).

Reactive oxygen metabolism-related antioxidants and antioxidant enzymes in the roots were measured at 0, 3, 12, 24, or 48 h. The enzymatic antioxidant activities of superoxide dismutase (SOD), catalase (CAT), peroxidase (POD), ASC peroxidase (APX), glutathione S-transferase (GST), glutathione reductase (GR), dehydroascorbate reductase (DHAR), and monodehydroascorbate reductase (MDHAR) were quantified according to the procedures described by Wu et al. (2017). For each enzyme assay, 100 mg of ground root tissue was homogenized in 1 mL of 50 mM sodium phosphate buffer (pH 7.0) containing 0.2 mM ethylenediaminetetraacetic acid (EDTA) and 1% (w/v) polyvinylpyrrolidone (PVP) on ice, and the homogenates were then centrifuged at 12,000 × g and 4°C for 20 min. The supernatants were subsequently used for the determination of enzyme activities. For CAT, POD, and SOD, absorbance was determined with a spectrophotometer (Spectronic Instruments, Rochester, NY, USA) at 240, 470, and 560 nm, respectively. For DHAR and APX, absorbance was measured using a spectrophotometer at 265 and 290 nm, respectively. For GST, GR, and MDHAR, absorbance was assayed using a spectrophotometer (Spectronic Instruments, Rochester, NY, USA) at 340 nm.

The non-enzymatic antioxidant contents of ASC, dehydroascorbate (DHA), glutathione (GSH), oxidized GSH (GSSG), and malondialdehyde (MDA) were measured. Frozen ground root powder (0.1 g) was homogenized with 5 mL of 5% ice-cold trichloroacetic acid (TCA), and the mixture was then centrifuged at 8,000 × g and 4°C for 10 min. The supernatant was immediately used for analysis. The contents of ASC, DHA, GSH and GSSG in the root samples were determined according the method described by Chen et al. (2011). The MDA contents in root samples were quantified according to the method described by Xu et al. (2016). All enzyme activities and non-enzymatic antioxidant contents in the roots were measured in three biological replicates. Each time point of the Rg_1_, Rg_1_+ASC, and Rg_1_+gentiobiose treatments was compared with each other, and the different time points of each treatment were compared with time 0 h.

### Statistical analysis

The data were analyzed via one-way analysis of variance and compared using *post-hoc* Duncan's test (*p* < 0.05) with PASW Statistics 18 (SPSS Inc.). The results of the cluster analysis of gene expression were evaluated using heatmap.2 in the “gplots” package in R (version 3.1.2).

## Results

### Rg_1_ concentrations in cultivated soil inhibit the growth of *P. notoginseng*

**Figure 1:**
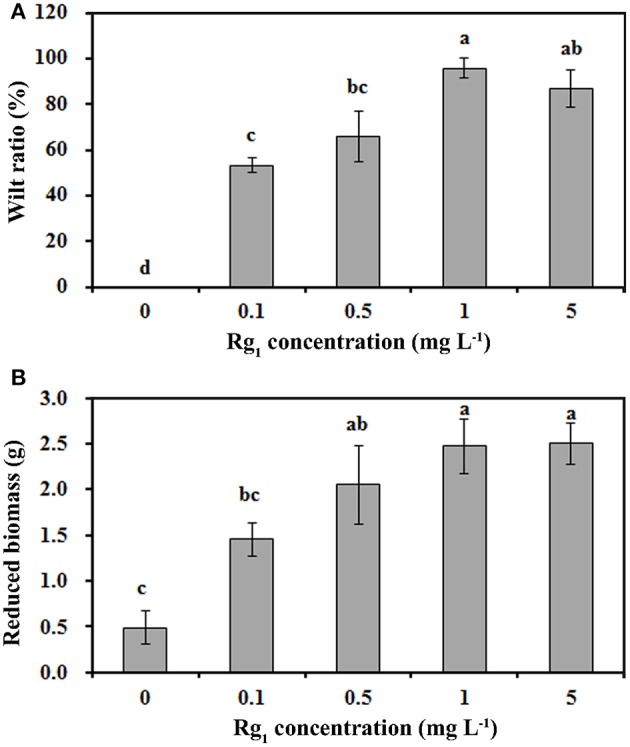
Rg_1_ inhibition of *P. notoginseng* seedling growth. **(A,B)** respectively, show the wilt ratio and the reduced biomass of seedlings after treatment with Rg_1_ at concentrations of 0.1, 0.5, 1.0, and 5.0 mg L^−1^. The values represent the means ± SE. The bars with different letters are significantly different (*p* < 0.05; *n* = 4).

The mean and range of Rg_1_ concentrations were 0.70 mg kg^−1^ and 0.21~1.32 mg kg^−1^, respectively, in the 1-year consecutively cultivated bulk soil, 1.09 mg kg^−1^ and 0.83~1.54 mg kg^−1^ in the 2-year consecutively cultivated bulk soil, and 1.41 mg kg^−1^ and 1.03~2.01 mg kg^−1^ in the 3-year consecutively cultivated bulk soil (Table S1). Thus, Rg_1_ concentrations of 0.1, 0.5, 1.0, and 5.0 mg L^−1^ were selected to test its autotoxicity during seedling growth. Compared with the control, the seedling wilt ratio (Figure 1A) and the reduction in biomass (Figure 1B) were increased by Rg_1_ treatment in a dose-dependent manner. The greatest values for the wilt ratio and the reduction in biomass were obtained when the seedlings were treated with Rg_1_ at a concentration of 1.0 mg L^−1^. Hence, this concentration was chosen for further analyses.

### Rg_1_ causes root cell death and cell wall degradation

**Figure 2:**
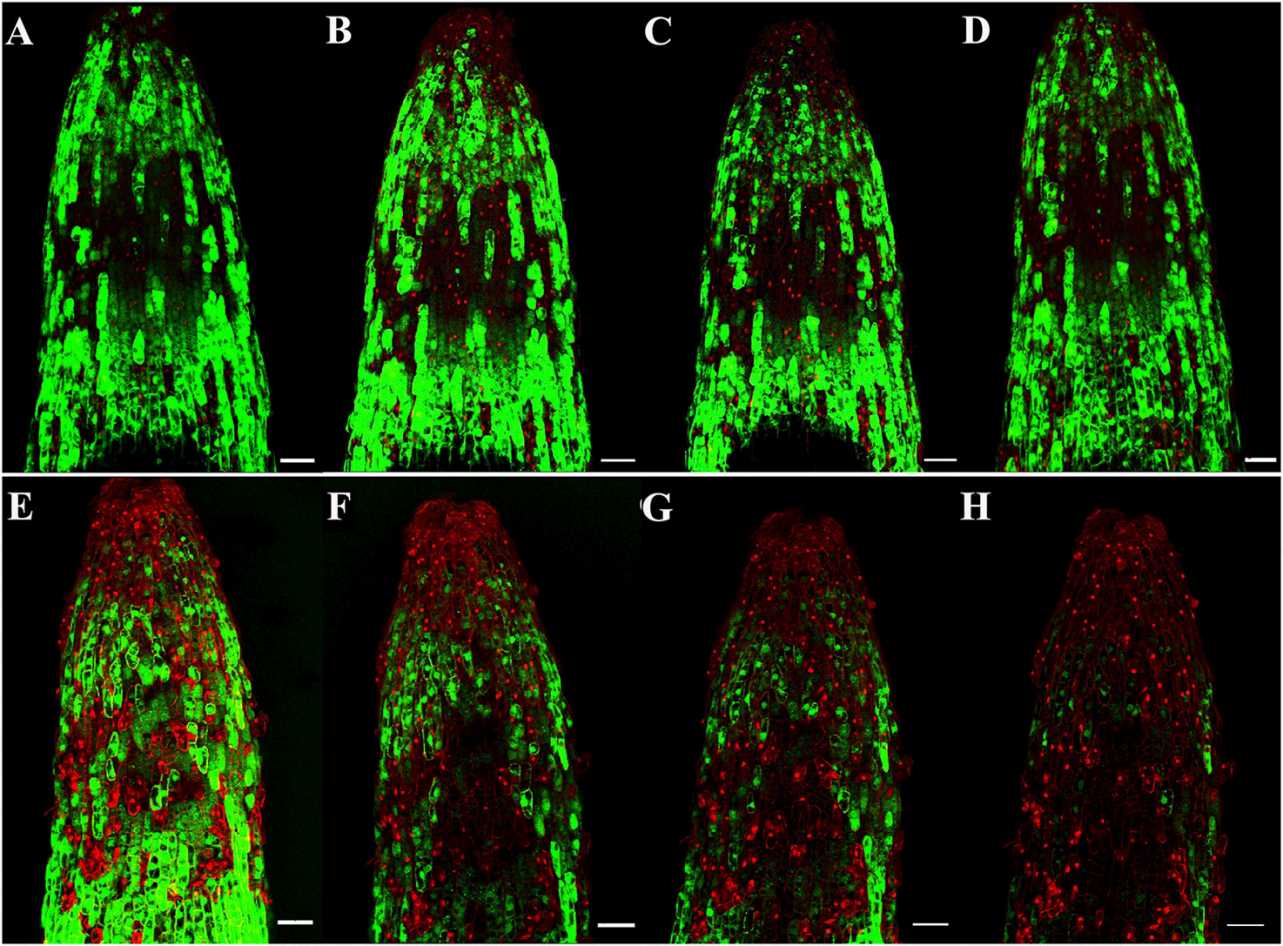
Progression of root cell death in *P. notoginseng* after treatment with Rg_1_ at a concentration of 1.0 mg L^−1^. Root cells were stained with FDA and PI. The photographs were taken at 0.5, 1.0, 2.0, and 3.0 h after treatment. Cells stained by FDA are viable (green), whereas cells stained by PI are dead (red). **(A–D)** Photographs of roots that were not exposed to Rg_1_ for 0.5, 1.0, 2.0, or 3.0 h, respectively; **(E–H)** photographs of roots treated with Rg_1_ for 0.5, 1.0, 2.0, or 3.0 h, respectively. The bars indicate 100 μm.

To identify the progression of Rg_1_-induced cell death in roots, we stained the roots with FDA-PI. FDA stains living cells green, and PI stains dead cells red. As shown in Figure 2, most of the apical and subapical root cells in the control treatment were sporadically stained with FDA (Figures 2A–D). However, in roots treated with 1.0 mg L^−1^ Rg_1_, PI-stained dead cells began to distinctly appear 0.5 h after treatment and were significantly increased after prolonged treatment. After 3 h of incubation, almost all root cells were strongly stained with PI (Figures 2E–H). When the roots of seedlings were immersed in water amended with different concentrations of Rg_1_, the conductivity of the hydroponic solution significantly increased in a dose-dependent manner (Figure S1).

**Figure 3:**
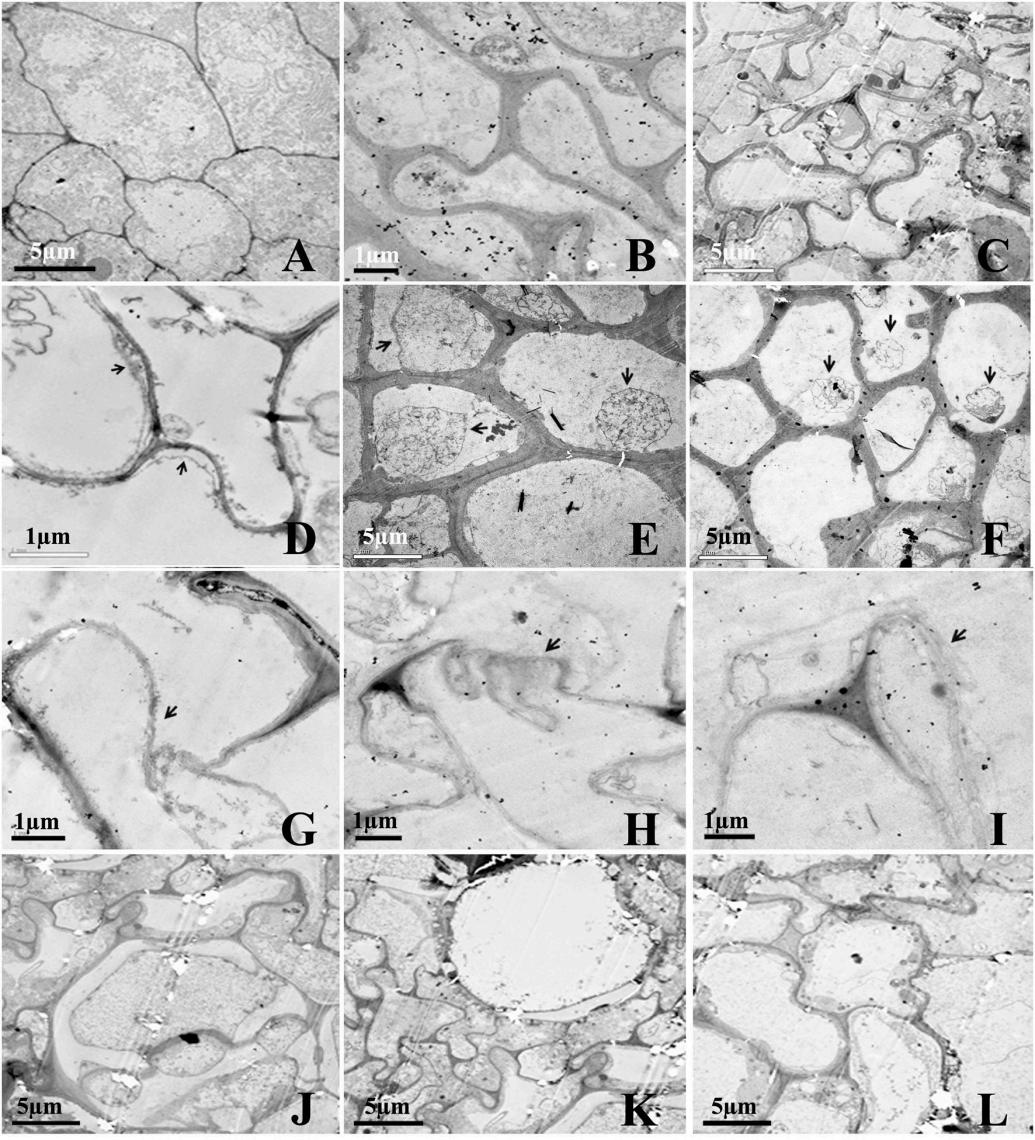
Transmission election micrographs of *P. notoginseng* root cells after treatment with Rg_1_. **(A)** Normal cells from the root tip, with nuclei, a plasma membrane, endoplasmic reticulum and mitochondria. **(B,C)** The cell wall subsequently began to thicken; the cell shape became distorted; and the cells shrunk (**B**, 3 h; **C**, 24 h). **(D–F)** The cytoplasm became concentrated, and plasmolysis appeared (arrows) (**D**, 3 h; **E**, 6 h; **F**, 12 h). **(G–I)** The cell wall was digested (arrows) (**G**, 12 h; **H** and **I**, 24 h). **(J,K)** The cell wall between two cells was digested, and large vacuoles subsequently appeared (**J–L**, 24 h).

The effect of Rg_1_ on root cell morphology was further observed via TEM. Cells from the apical meristem of untreated roots exhibited few small vacuoles with large, round nuclei and intact organelles (Figure 3A). However, the addition of 1.0 mg L^−1^ Rg_1_ to the roots led to many abnormalities. After treatment from 3 h (Figure 3B) to 24 h (Figure 3C), the cell wall began to thicken, the shape of the cells became distinctly distorted, and the cells began to shrink. As the treatment time increased, the plasma membrane retracted from the cell wall, causing plasmolysis (Figure 3D), and the cytoplasm became concentrated (Figures 3E,F). Furthermore, cellular structures broke down, including digestion of the cell wall (Figures 3G–I) and disappearance of various organelles, and vacuoles occupied most of the space in the cells (Figures 3J–L).

### Transcriptome profiles of the root response to Rg_1_ treatment

To obtain the reference *P. notoginseng* transcriptome for the lateral roots, an RNA-Seq library was constructed using RNA from all root samples. A total of 48.95 G nucleotides with a Q_20_ percentage of 94.39% were generated. The Trinity package assembled 10,0125 unigenes, with a mean size of 631.82 bp (Table S2). A total of 100,125 unigenes were successfully annotated in at least one of the NR, NT, Swiss-Prot, KEGG, GO, and COG databases, and 16,601 unigenes (16.58%) were annotated in all six databases (Figure S2).

**Figure 4:**
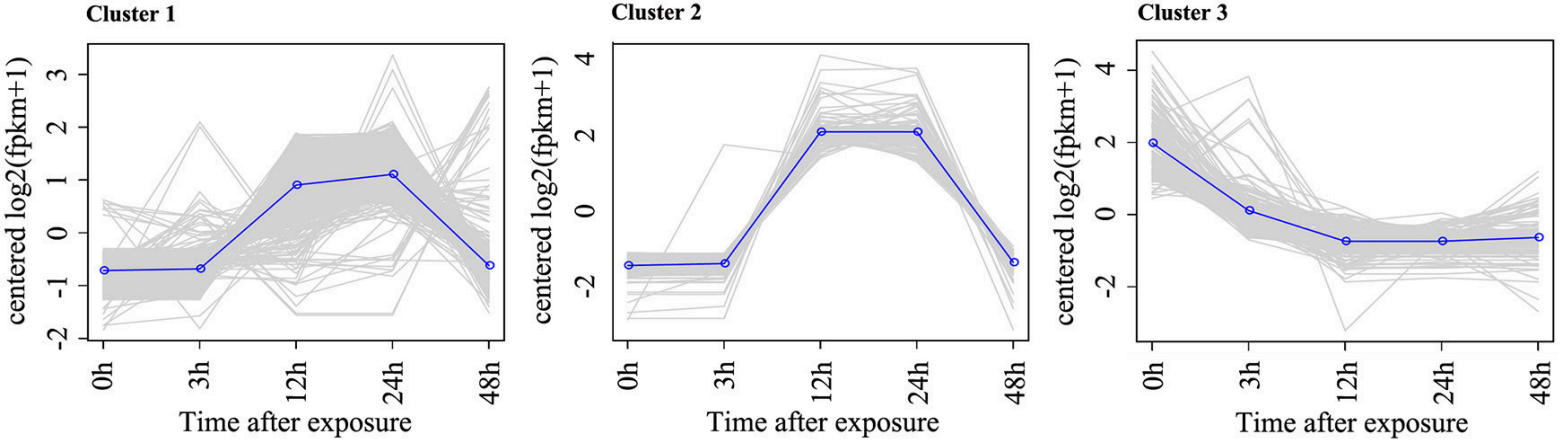
Representative time-course profile of gene expression clusters. For each time point and each gene, the log_2_ (FPKM+1) value is shown to indicate the mean expression profile for each cluster. Gene expression cluster analysis was performed using STEM algorithms.

To identify the DEGs in response to Rg_1_, pairwise comparisons using 0 h as the control and 3, 12, 24, and 48 h as the treatments were performed. After 3, 12, 24, or 48 h of exposure to Rg_1_, we identified 5, 38, 1105, and 35 DEGs, respectively, compared with the 0-h treatment (Table S3). All DEGs were separated into three significant clusters (*p* < 0.001) based on similarities in the expression profiles (Figure 4). Some DEGs involved in plant growth and development, including oxidative phosphorylation, ribosomes, and RNA transport, were significantly down-regulated after expose to Rg_1_ for 12 or 24 h (Table S4).

**Figure 5:**
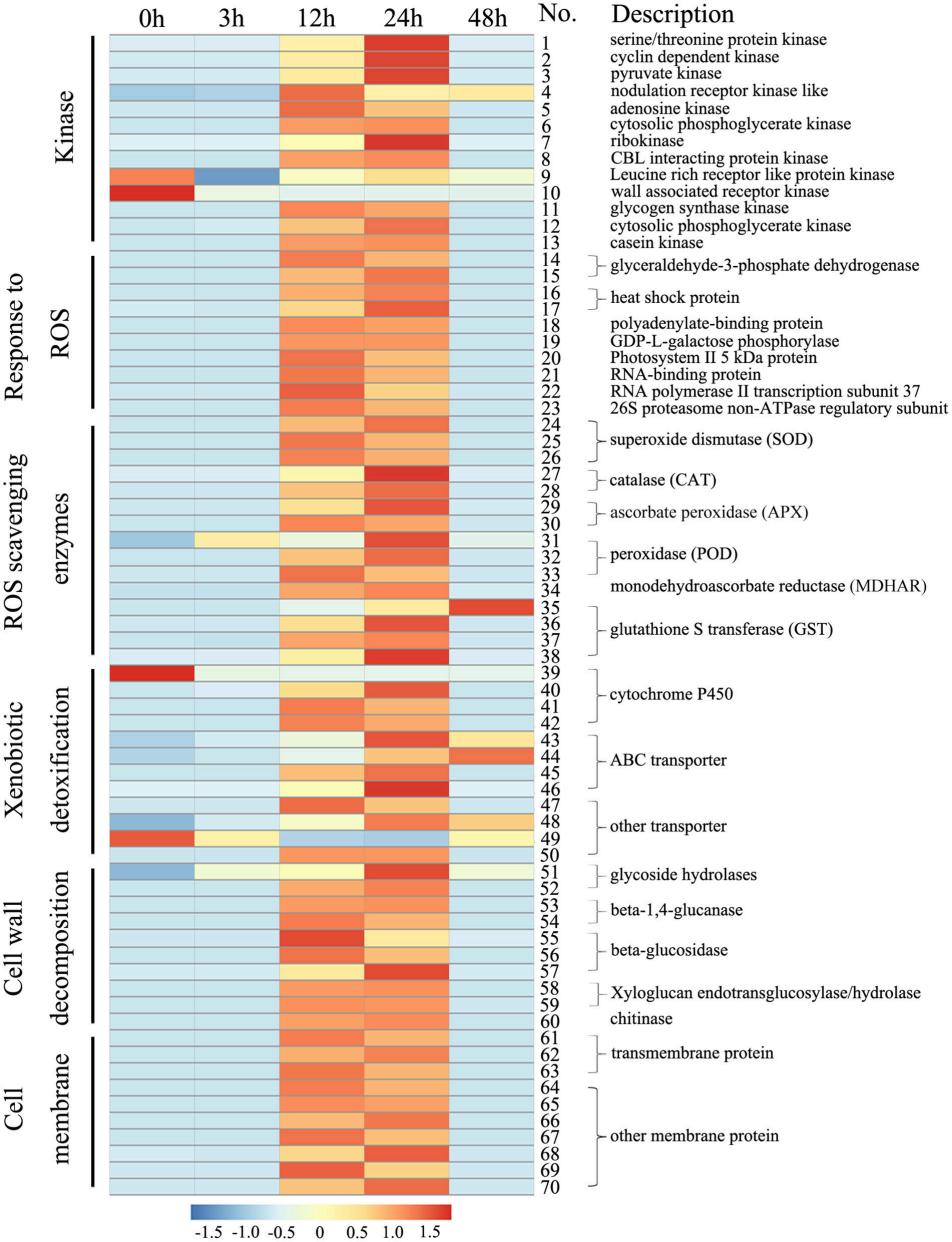
Heat maps of the differentially expressed genes in root cells involved in cell wall degradation or encoding ROS scavenging enzymes, ABC transporters, transmembrane proteins and protein kinases after exposure to Rg_1_ for 0, 3, 12, 24, and 48 h. Detailed descriptions of these genes are provided in Table S5.

Based on the cellular data, we further analyzed the transcript profiles of genes involved in ROS metabolism and cell wall- and cell membrane-related processes (Figure 5; Table S5). ROS metabolism-related genes, including 10 ROS response genes and 15 ROS scavenging enzymes genes (i.e., SOD, CAT, APX, POD, MDHAR, and GST), were significantly up-regulated by Rg_1_ treatment for 12 or 24 h. For example, one GST gene (unigene 0072579), which encodes a type of antioxidative enzyme involved in the ASC-GSH cycle, was significantly up-regulated 100- and 114-fold by Rg_1_ treatment for 12 and 24 h, respectively, compared with the level detected at 0 h. Cell wall decomposition-related genes, including two glycoside hydrolase genes, two beta-1,4-glucanase genes, three beta-glucosidase genes, two xyloglucan endotransglucosylase/hydrolase genes, and one chitinase gene, were significantly up-regulated in the roots after exposure to Rg_1_ for 12 or 24 h. For example, one glycoside hydrolase gene (unigene 0014244) involved in cell wall degradation was up-regulated 1.94-, 2.21-, and 3.67-fold by Rg_1_ treatment for 3, 12, and 24 h, respectively. In addition, 10 membrane protein-related genes were significantly up-regulated, and many genes encoding proteins involved in xenobiotic detoxification (i.e., cytochrome P450, ABC transporters, and other transporters), protein kinases, and transcription factors were also up-regulated (Figure 5; Table S5).

### ROS accumulation is induced by Rg_1_ but blocked by exogenous antioxidants

**Figure 6:**
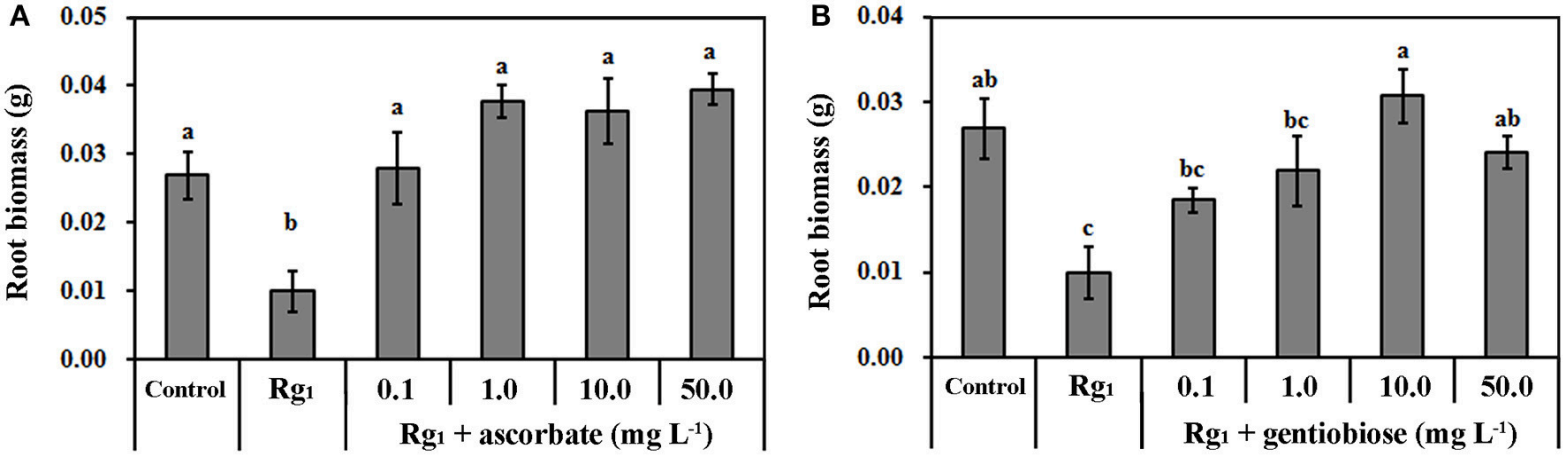
Effect of Rg_1_ with or without ascorbate **(A)** or gentiobiose **(B)** on root biomass. The values represent the means ± SE. The bars with different letters are significantly different (*p* < 0.05; *n* = 4).

The transcriptomic results demonstrated that Rg_1_ might disturb ROS metabolism in roots and cause cell death. To test this hypothesis, we further studied the effect of Rg_1_, with or without the exogenous antioxidants ASC and gentiobiose, on root growth and ROS metabolism. Ascorbate is an antioxidant involved in the ASC-GSH cycle (Gill and Tuteja, 2010). Gentiobiose (6-O-β-D-glucopyranosyl-D-glucose) is reported to up-regulate the synthesis of GSH and activate the ASC-GSH cycle (Takahashi et al., 2014). Root biomass was reduced by 63.20% when seedlings were grown on media amended with Rg_1_ at a concentration of 1.0 mg L^−1^ compared with the control (Figure 6). However, the toxicity of Rg_1_ to root growth was significantly alleviated to the control levels by the addition of ASC at concentrations of 1.0, 10.0, and 50.0 mg L^−1^ (Figure 6A) or gentiobiose at a concentration of 10.0 or 50.0 mg L^−1^ (Figure 6B). Treatment with ASC or gentiobiose alone at concentrations from 0.1 to 50 mg L^−1^ did not show significant toxicity to plants, as indicated by the root biomass (Figure S3).

**Figure 7:**
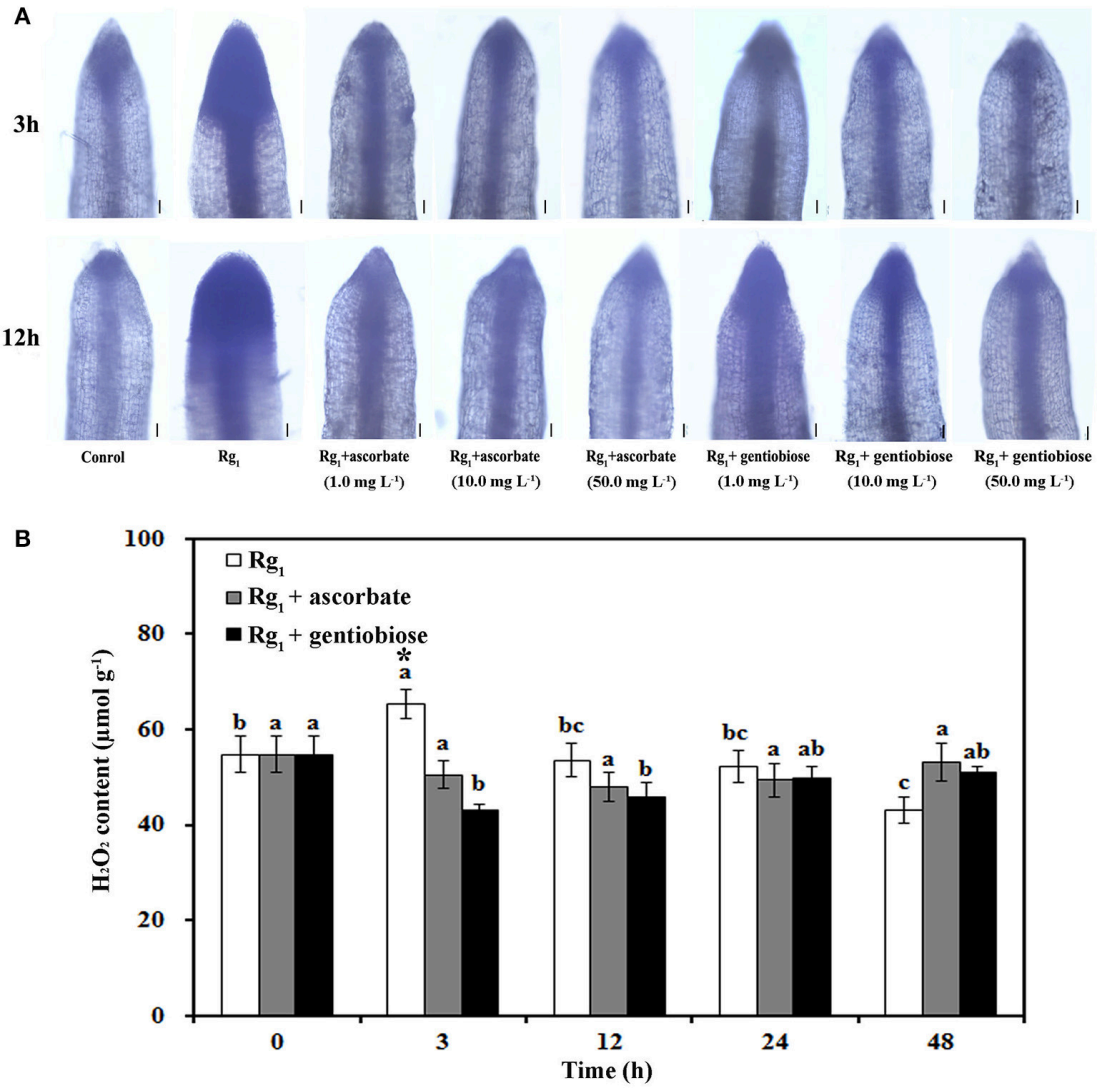
Effect of Rg_1_ with or without exogenous antioxidants on ROS accumulation. **(A)** Superoxide (${\text{O}}_{2\cdot }^{-}$) levels in root tips after treatment with Rg_1_ (1.0 mg L^−1^) with or without ascorbate or gentiobiose at concentrations ranging from 0.0 to 50.0 mg L^−1^. Superoxide was assessed by *in situ* staining with NBT. Bar = 50 μm. **(B)** Content of H_2_O_2_ in roots after treatment with Rg_1_ (1.0 mg L^−1^) with or without ascorbate (1.0 mg L^−1^) or gentiobiose (10.0 mg L^−1^). The values represent the means ± SE. Different letters on the bars indicate significant differences between different treatment durations (*p* < 0.05; *n* = 3). An asterisk (*) indicates that the differences between the Rg_1_, Rg_1_+ascorbate, and Rg_1_+gentiobiose treatments at the same time were significant at *p* < 0.05.

To elucidate the effect of Rg_1_ treatment on ROS metabolism over time in roots, the status of antioxidants and antioxidant enzymes was measured. ${\text{O}}_{2\cdot }^{-}$ significantly accumulated in the roots after exposure to Rg_1_ for 3 and 12 h (Figure 7A). However, the level of ${\text{O}}_{2\cdot }^{-}$ in the roots was reduced by the addition of the antioxidants ASC and gentiobiose (Figure 7A). Significant accumulation of H_2_O_2_ was measured after 3 h of Rg_1_ treatment, and this level was decreased after 12 h of treatment (Figure 7B). Roots that were simultaneously treated with Rg_1_ and either ASC (1.0 mg L^−1^) or gentiobiose (10.0 mg L^−1^) showed significantly reduced H_2_O_2_ accumulation compared with the plants treated with Rg_1_ alone. The lowest H_2_O_2_ content was observed after gentiobiose treatment for 3 h (Figure 7B).

**Figure 8:**
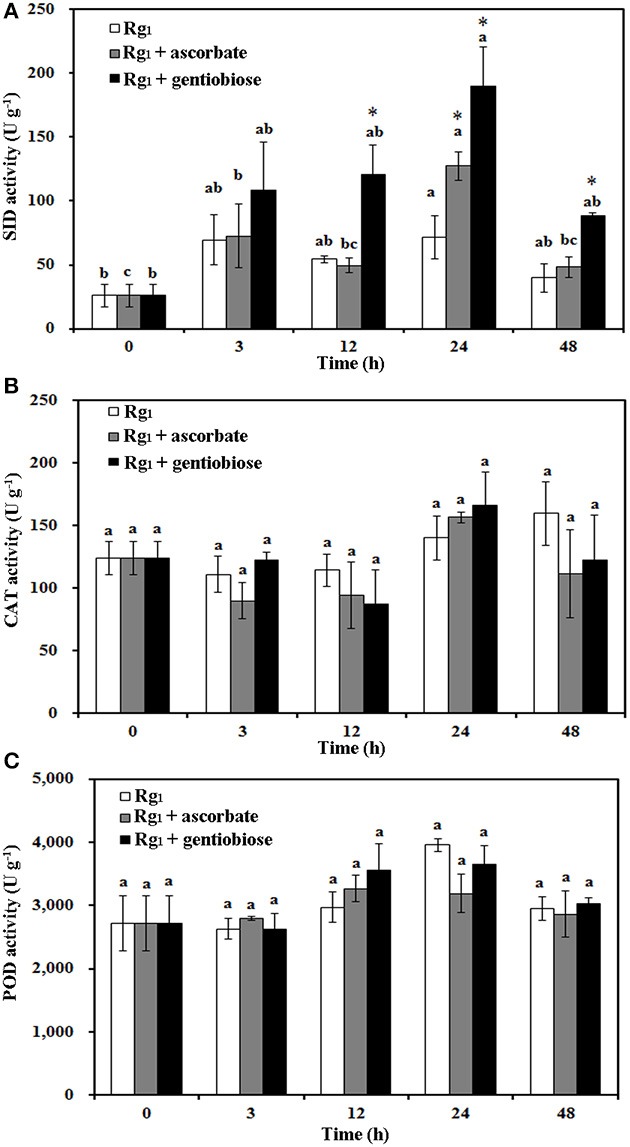
Effect of Rg_1_ (1.0 mg L^−1^) with or without ascorbate (1.0 mg L^−1^) or gentiobiose (10.0 mg L^−1^) on antioxidative enzyme activity in roots over 48 h of treatment. **(A)** SOD activity. **(B)** CAT activity. **(C)** POD activity. Each bar represents the mean ± SE of three independent experiments. Different letters on the bars indicate significant differences between different treatment durations (*p* < 0.05). An asterisk (*) indicates that the differences between the Rg_1_, Rg_1_ + ascorbate, and Rg_1_ + gentiobiose treatments at the same time was significant at *p* < 0.05.

**Figure 9:**
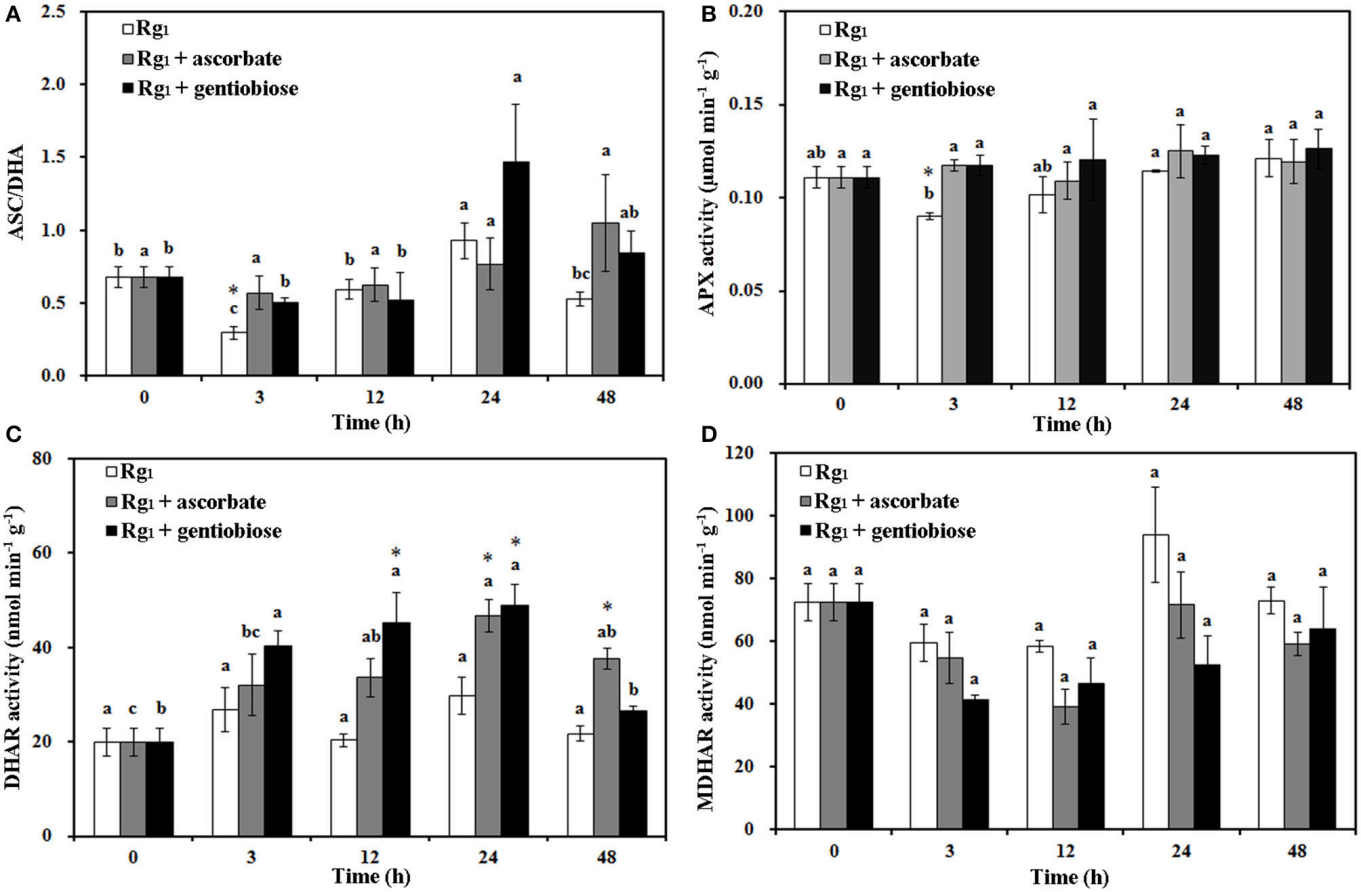
Effect of Rg_1_ (1.0 mg L^−1^) with or without ascorbate (1.0 mg L^−1^) or gentiobiose (10.0 mg L^−1^) on ascorbate (ASC)/dehydroascorbate (DHA) **(A)**, ascorbate peroxidase (APX) **(B)**, dehydroascorbate reductase (DHAR) **(C)**, and monodehydroascorbate reductase (MDHAR) **(D)** activities in roots over 48 h of treatment. Each bar represents the mean ± SE of three independent experiments. Different letters on the bars indicate significant differences between different treatment durations (*P* < 0.05). An asterisk (*) indicates that the differences between the Rg_1_, Rg_1_ + ascorbate, and Rg_1_ + gentiobiose treatments at the same time were significant at *p* < 0.05.

**Figure 10:**
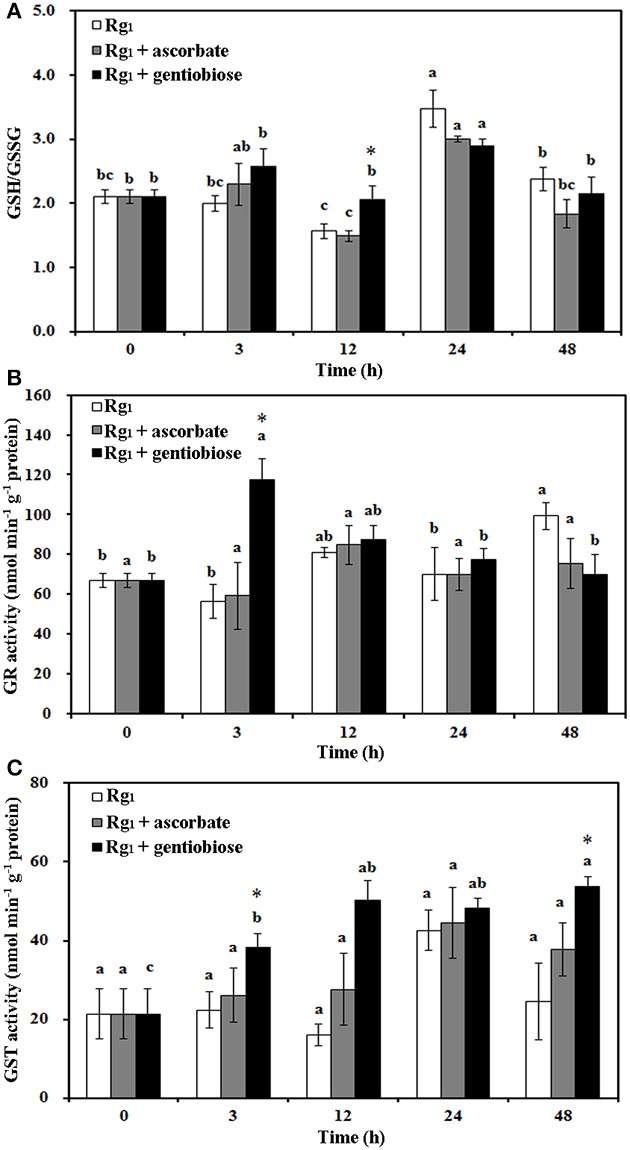
Effect of Rg_1_ (1.0 mg L^−1^) with or without ascorbate (1.0 mg L^−1^) or gentiobiose (10.0 mg L^−1^) on the ratio of reduced glutathione (GSH) to oxidized glutathione (GSSG) **(A)** and the activities of glutathione reductase (GR) **(B)** and glutathione S-transferase (GST) **(C)** in roots over 48 h of treatment. Each bar represents the mean ± SE of three independent experiments. Different letters on the bars indicate significant differences between different treatment time (*p* < 0.05). An asterisk (*) indicates that the differences between the Rg_1_, Rg_1_ + ascorbate, and Rg_1_ + gentiobiose treatments at the same time were significant at *p* < 0.05.

Under Rg_1_ stress conditions, some root antioxidant enzymes were also significantly affected. Root SOD activity was significantly induced by Rg_1_ treatment, and significantly higher SOD activity was observed in roots after exposure to Rg_1_ plus gentiobiose treatment for 12, 24, and 48 h and after exposure to Rg_1_ plus ASC treatment for 24 h (Figure 8A). CAT and POD activities were slightly increased, albeit not significantly, by treatment with Rg_1_ and the antioxidants (Figures 8B,C). The antioxidants and enzymes involved in the ASC-GSH cycle were also affected by Rg_1_ treatment. The ASC/DHA ratio and APX activity in roots were significantly decreased after exposure to Rg_1_ for 3 h (Figures 9A,B; Figures S4A,B). Treatment with either exogenous ASC or gentiobiose resulted in resumption of APX activity, increased the ASC/DHA ratio and even stimulated the activity of DHAR (Figure 9C). However, MDHAR activity was not significantly affected by treatment with Rg_1_ or the antioxidants (Figure 9D). The GSH/GSSG ratio also decreased after exposure to Rg_1_ for 12 h (Figure 10A; Figures S4C,D) but significantly increased after the addition of gentiobiose (Figure 10A). Root GR and GST activities were also significantly increased by the exogenous addition of gentiobiose (Figures 10B,C).

## Discussion

*P. notoginseng* shows severe autotoxicity in continuous cropping lands (Yang et al., 2015). It is critically important to decipher the response of plants to autotoxins in order to overcome the problem of autotoxicity. Our previous studies demonstrated that the allelochemical ginsenoside Rg_1_ is a specific autotoxin that inhibits seedling emergence and growth in *P. notoginseng* (Yang et al., 2015). In this study, we found that Rg_1_ at concentrations found in cultivated soil could induce over-accumulation of ROS, which can cause oxidative damage to cells and has been indicated as a direct or indirect effect of exposure to a number of allelochemicals in plant cells (Kobayashi et al., 2002; Yu et al., 2003; Lara-Nuñez et al., 2006; Chi et al., 2013; Wu et al., 2015). Here, Rg_1_ induced over-accumulation of ROS (${\text{O}}_{2\cdot }^{-}$ and H_2_O_2_) in root cells through suppression of the ASC/GSH cycle, which subsequently disrupted the integrity of the cell membrane, ultimately damaged root cells, and inhibited root growth.

Rg_1_ induced over-accumulation of ROS (${\text{O}}_{2\cdot }^{-}$ and H_2_O_2_) by affecting the SOD enzyme and the ASC-GSH cycle. RNA-Seq analysis demonstrated that ROS response genes and ROS scavenging enzyme genes were up-regulated after the roots were exposed to Rg_1_. These data implied that Rg_1_ interfered with the process of ROS metabolism. ROS signaling plays a vital role in plant defense against multiple stress stimuli. However, ROS over-accumulation is toxic to cell growth (Baxter et al., 2014). Plant cells employ a series of antioxidant substances (i.e., ASC and GSH) or enzymes (SOD) to dismutate ${\text{O}}_{2\cdot }^{-}$, whereas CAT, peroxidase cycle enzymes (POD), or ASC-GSH cycle-related enzymes (i.e., APX, MR, and GR) are employed to reduce H_2_O_2_ (Gill and Tuteja, 2010). In this study, over-accumulation of ${\text{O}}_{2\cdot }^{-}$ was observed in root cells after exposure to Rg_1_. The gene transcript levels and enzyme activity of SOD were simultaneously enhanced to transform ${\text{O}}_{2\cdot }^{-}$ into H_2_O_2_. Although the gene transcription and enzyme activities of CAT and POD were also slightly induced, the level of enzyme activity was not significant, suggesting that alteration of CAT and POD activities is not part of the response to H_2_O_2_ over-accumulation due to Rg_1_. The decreased content of the antioxidant ASC in the ASC-GSH cycle in root cells after exposure to Rg_1_ for 3 h might imply a decreased ability to convert H_2_O_2_ to H_2_O. Ascorbate, which is involved in the ASC-GSH cycle, acts as a natural antioxidant to protect cellular components from radical damage (Beyer, 1994; Green and Fry, 2005). The levels of ASC-GSH cycle metabolites are often elevated to scavenge ROS during the exposure of plant cells to abiotic stress (Navari-Izzo et al., 1997; Jiang et al., 2007). Indeed, we observed a high level of H_2_O_2_ accumulation in the roots after exposure to Rg_1_ for 3 h (Figure 7). In the ASC-GSH cycle, a decrease in either the ASC/DHA or GSH/GSSG ratio is considered a determinant of oxidative stress (Shi et al., 2015; Marta et al., 2016). When roots were exposed to Rg_1_ for 3 or 12 h, the ASC/DHA and GSH/GSSG ratios were significantly decreased (Figures 9, 10). In particular, a significant decrease in the activity of APX (Figure 9), which uses ASC as a reductant to scavenge H_2_O_2_ (Bose et al., 2014), was observed. With the prolongation of Rg_1_ stress for 12 or 24 h, the transcript levels of ASC-GSH cycle-related antioxidative enzymes (i.e., GST and MDHAR) increased. It appears that the activities of these enzymes are increased in root cells in an attempt to counteract the harmful effects of ROS during the later stages of the effects of Rg_1_. This finding further confirmed that Rg_1_ could enhance ROS accumulation in roots through suppression of the ASC-GSH cycle to scavenge H_2_O_2_ into H_2_O and O_2_. When the antioxidants ASC and gentiobiose were exogenously added, the activity of ASC-GSH cycle enzymes (i.e., APX, DHAR, GR, and GST) and the content of antioxidants (i.e., ASC and GSH) were significantly increased, and Rg_1_ autotoxicity was alleviated (Figures 9, 10). These data also implied that exogenous application of antioxidants might be a potential strategy for overcoming the problem of autotoxicity and thus an alternative to the strategies of microbial degradation and activated charcoal adsorption (DŽafić et al., 2013; Li et al., 2014; Yang et al., 2015).

ROS over-accumulation in response to allelochemicals damages cells, often increasing membrane permeability and causing generalized cellular disruption that ultimately leads to cellular damage and death (Lin et al., 2000; Zeng et al., 2001; Yu et al., 2003). Our previous work demonstrated that autotoxic ginsenosides cause death of cells distributed in the apical and subapical roots of *P. notoginseng* (Yang et al., 2015). In the present study, a RNA-Seq analysis further demonstrated that some genes involved in plant growth and development, including genes related to the ribosomes, photosynthesis, RNA transport and oxidative phosphorylation, were down-regulated after the roots were exposed to Rg_1_ (Table S4), which could explain the growth inhibition effects of Rg_1_ on *P. notoginseng* at the transcriptome level. Cellular data and transcriptome analyses further demonstrated that Rg_1_ altered the membrane permeability and cell wall structure, which ultimately caused root cell death (Figure 3).

Rg_1_ disrupted the cell membrane permeability. FDA-PI staining demonstrated that the number of dying or dead cells was increased in the roots after exposure to Rg_1_ (Figure 2). The staining of Rg_1_-treated root cells with PI suggested that the membranes of the dead cells were permeable because PI can only penetrate cells with permeable membranes (i.e., dead cells; Fan et al., 2013). This finding implied that the cell membrane structure might be destroyed by Rg_1_. RNA-Seq analysis further demonstrated that 10 membrane protein-related genes were significantly up-regulated after exposure to Rg_1_ for 12 or 24 h. Among these genes, transmembrane protein 214-A isoform 1 has been reported to mediate endoplasmic reticulum stress-induced apoptosis (Li et al., 2013), and up-regulation of this gene results in destruction of the cell membrane. The increased conductivity of the hydroponic seedling solution further confirmed the presence of permeable membranes after exposure to Rg_1_ (Figure S1).

Cell ultrastructural observations demonstrated that Rg_1_ treatment resulted in obvious degradation of the root cell wall. Consistent with this finding, transcriptomic analyses further confirmed that cell wall decomposition-related genes, including glycoside hydrolases, beta-1,4-glucanase, beta-glucosidase, xyloglucan endotransglucosylase/hydrolase, and chitinase, were induced after exposure to Rg_1_ for 12 or 24 h (Figure 5). Beta-1,4-glucanase and beta-glucosidase are key enzymes in the hydrolysis of cellulose into cellobiose or sophomores (Maclachlan and Brady, 1994). Xyloglucan endotransglucosylase/hydrolase is the enzyme responsible for cutting and re-joining intermicrofibrillar xyloglucan chains, which causes wall loosening (Fry et al., 1992). O-Glycosyl hydrolase family 17 proteins can hydrolyse the polysaccharides present in the cell walls of plants (Henrissat et al., 1998). Chitinases can hydrolyse the N-acetylglucosamine polymer chitin in plant tissues (Punja and Zhang, 1993). Collectively, these data demonstrated that the cell wall might be an important target of Rg_1_ damage involved in cell death. Other studies have also demonstrated that many cell wall-targeting agents, such as peptides, echinocandins and bleomycin-Fe(II), can induce the accumulation of ROS, leading to cell necrosis and ultimately resulting in cell wall disruption (Lim et al., 1995; Denness et al., 2011; Maurya et al., 2011; Ramirez-Quijas et al., 2015). Our study is one of the first to show that increases in ROS induced by allelochemical autotoxins can result in cell wall degradation.

## Conclusions

**Figure 11:**
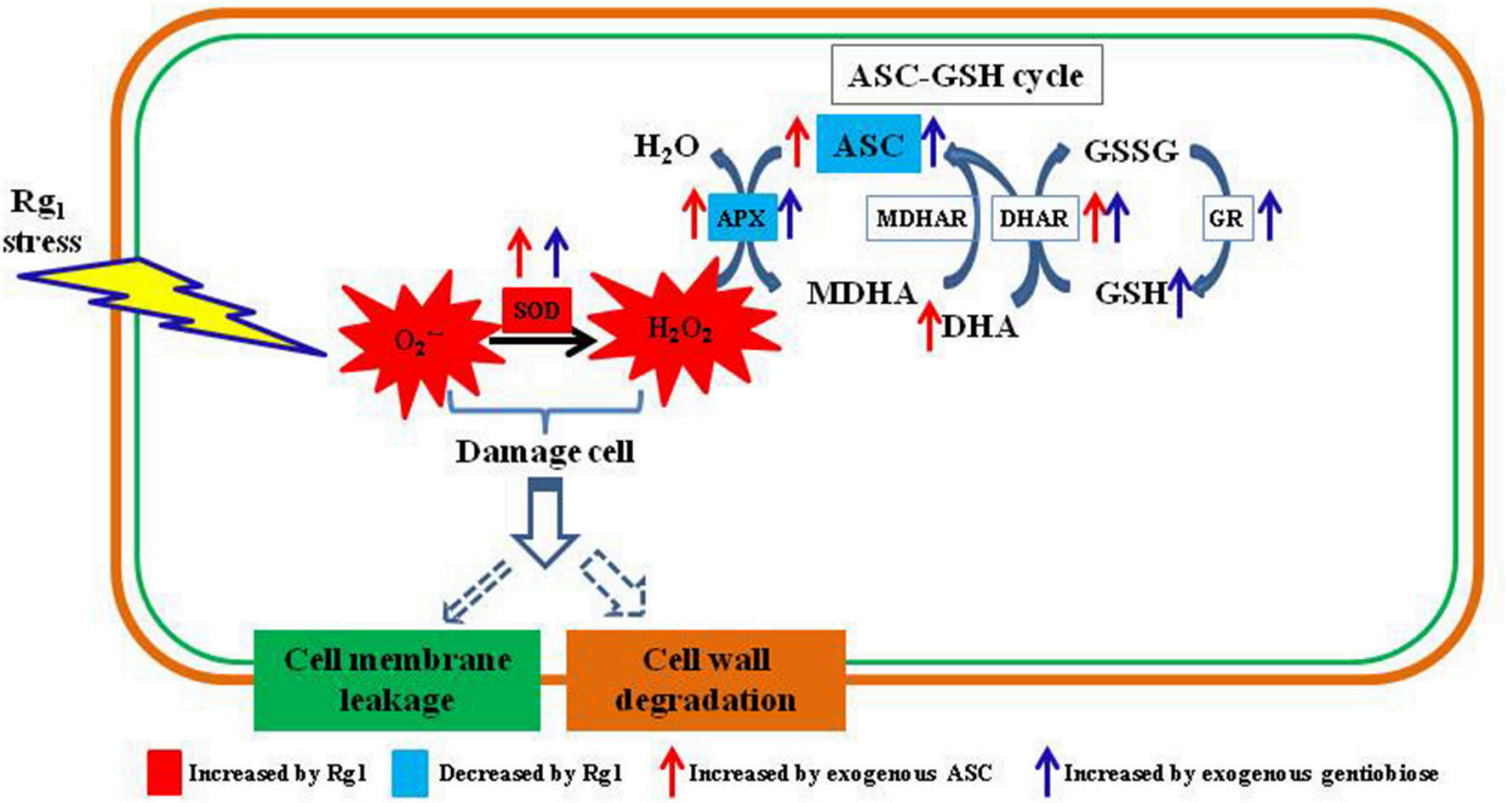
Schematic diagram showing autotoxin Rg_1_-induced root cell ROS accumulation, oxidative damage to cell wall and ASC-GSH cycle-related antioxidants and enzymes involved in ROS scavenging. ASC, reduced ascorbic acid; APX, ascorbate peroxidase; DHA, dehydroascorbic acid; DHAR, dehydroascorbate reductase; MDHAR, monodehydroascorbate reductase; GSH, glutathione; GSSG, oxidized glutathione; GR, glutathione reductase; SOD, superoxide dismutase.

In conclusion, Rg_1_ can damage the root cell membranes and cell walls of *P. notoginseng* by accelerating ROS (${\text{O}}_{2\cdot }^{-}$and H_2_O_2_) accumulation via suppression of APX enzyme activity and the contents of antioxidants (ASC and GSH) involved in the ASC-GSH cycle (Figure 11). Exogenous antioxidants (ASC and gentiobiose) may help root cells scavenge over-accumulated ROS resulting from Rg_1_ stress by promoting SOD activity and increases in ASC-GSH cycle enzymes (APX, DHAR, and GR) as well as non-enzymatic antioxidants (ASC and GSH). These findings implied that exogenous application of antioxidants could potentially overcome the problem of autotoxicity in agricultural production. Nevertheless, additional studies are needed to elucidate the regulatory mechanism and signaling pathways of Rg_1_ associated with antioxidant enzymes and genes. The transcriptome data indicated an array of changes in Rg_1_ stress-related genes, such as genes encoding protein kinases, transcription factors, and transporters, and thus provide a framework for further genetic studies on this phenomenon.

## Author contributions

SZ and MY conceived the study and directed the project. MY, YC, and JL performed the cellular activity test. CG, YX, XM, and HH performed the transcriptome sequencing, assembly, and analyses. YL and XH participated in the sample collection and sensitivity testing. All authors participated in discussions and provided suggestions for manuscript improvement. SZ, MY, and YC wrote the paper with input from all authors.

### Conflict of interest statement

The authors declare that the research was conducted in the absence of any commercial or financial relationships that could be construed as a potential conflict of interest.

## Supplementary material

Figure S1 Changes in conductivity in hydroponic solution after the seedling roots of *P. notoginseng* were exposed to Rg_1_ for different treatment durations.

Figure S2 Number of unigenes annotated by BLASTX with an E-value threshold of 10^−5^ against protein databases.

Figure S3 Effects of treatment with ascorbate **(a)** or gentiobiose **(b)** alone at concentrations of 0.1–50 mg/L on plant root biomass.

Figure S4 Effects of Rg_1_ (1.0 mg L^−1^) with or without ascorbate (1.0 mg L^−1^) or gentiobiose (10.0 mg L^−1^) on ascorbate (ASC), dehydroascorbate (DHA), reduced glutathione (GSH), and oxidized glutathione (GSSG) activities in roots over 48 h of treatment. Each bar represents the mean ± SE of three independent experiments. Different letters on the bars indicate significant differences between different treatment durations (*p* < 0.05). An asterisk indicates that the differences between the Rg_1_, Rg_1_ + ascorbate, and Rg_1_ + gentiobiose treatments at the same time were significant at *p* < 0.05.

Table S1 Rg_1_ concentrations in soil in which *Panax notoginseng* was cultivated for 1–3 years.

Table S2 Summary of Illumina paired-end sequencing and assembly of root genes in *Panax notoginseng*.

Table S3 All differentially expressed genes identified after exposure to Rg_1_ for 0, 3, 12, 24, and 48 h.

Table S4 Pathway enrichment analysis of DEGs in different treatments.

Table S5 Differentially expressed unigenes related to the response to ROS, ROS scavenging enzymes, cell wall decomposition, the cell membrane, xenobiotic detoxification, transcription factors, and protein kinases.
