# Supplementary figures and images for: Panax notoginseng Root Cell Death Caused by the Autotoxic Ginsenoside Rg1 Is Due to Over-Accumulation of ROS, as Revealed by Transcriptomic and Cellular Approaches
Source: Front Plant Sci. 2018 Feb 28;9:264. doi: 10.3389/fpls.2018.00264 (PMC5836058; doi:10.3389/fpls.2018.00264)

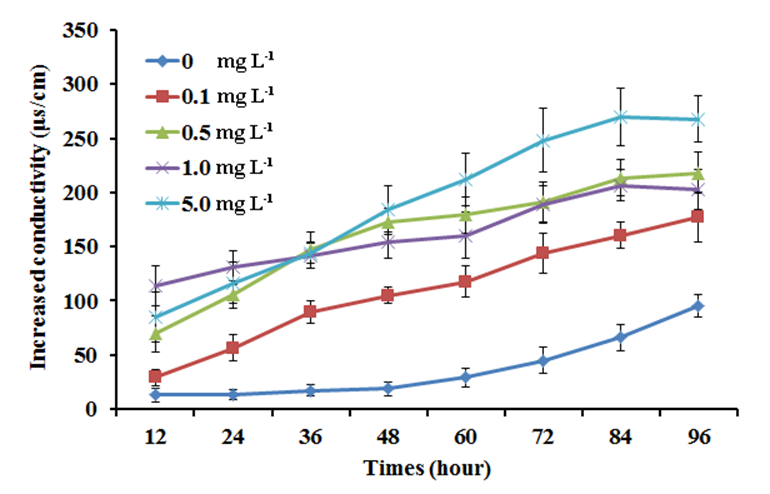

Supplement: Figure S1 — Changes in conductivity in hydroponic solution after the seedling roots of P. notoginseng were exposed to Rg1 for different treatment durations. [file Image1.TIF]

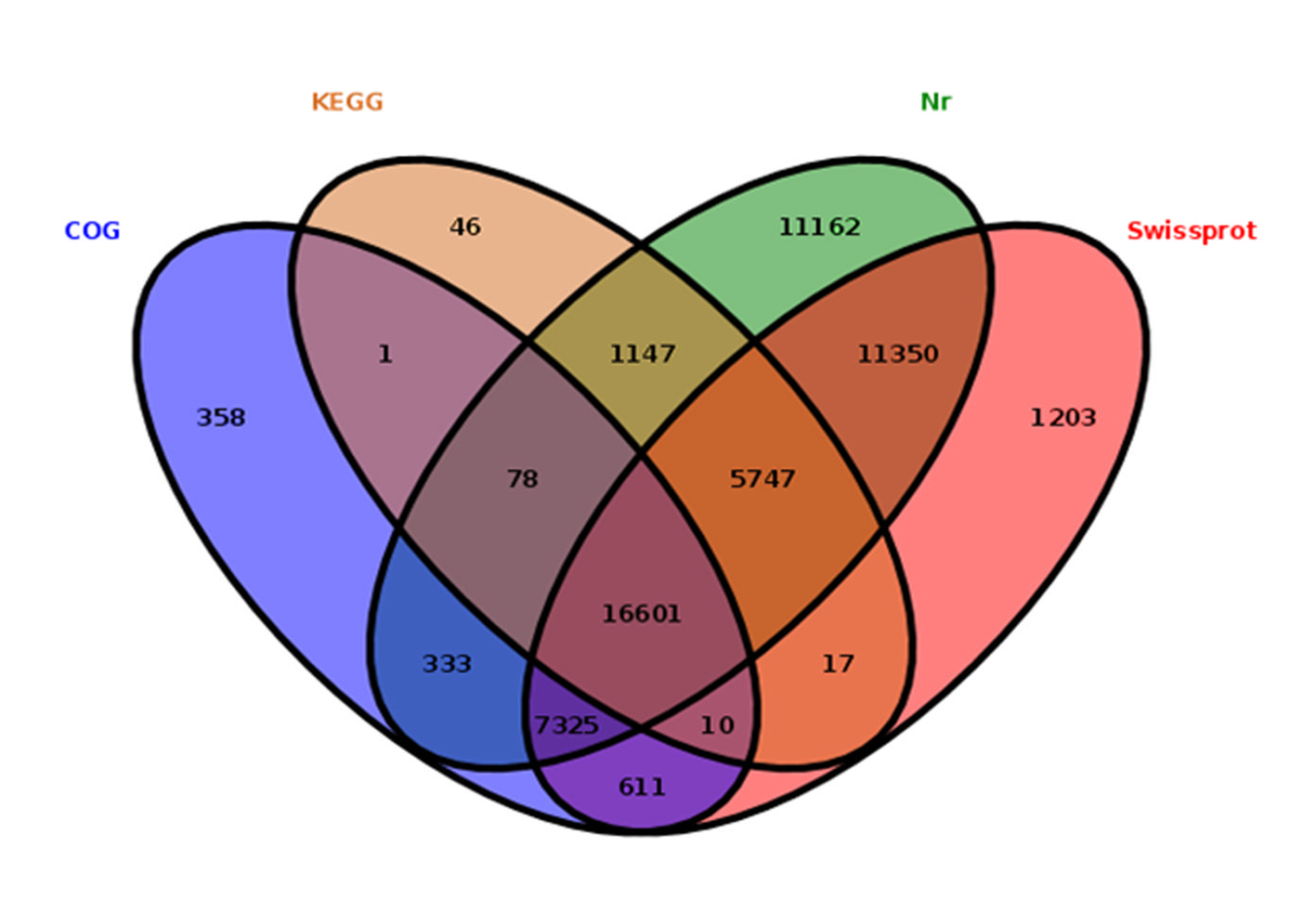

Supplement: Figure S2 — Number of unigenes annotated by BLASTX with an E-value threshold of 10−5 against protein databases. [file Image2.TIF]

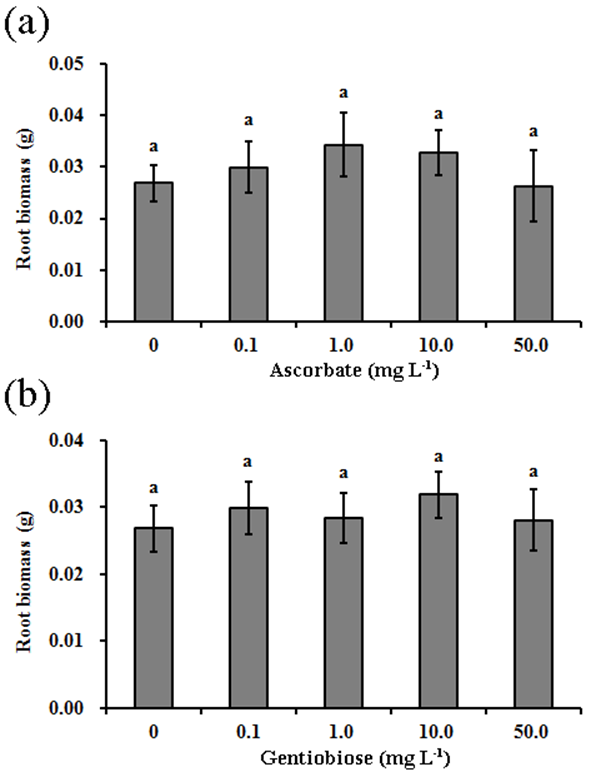

Supplement: Figure S3 — Effects of treatment with ascorbate (a) or gentiobiose (b) alone at concentrations of 0.1–50 mg/L on plant root biomass. [file Image3.TIF]
