# Supplementary material for: Panax notoginseng Root Cell Death Caused by the Autotoxic Ginsenoside Rg1 Is Due to Over-Accumulation of ROS, as Revealed by Transcriptomic and Cellular Approaches
Source: Front Plant Sci. 2018 Feb 28;9:264. doi: 10.3389/fpls.2018.00264 (PMC5836058; doi:10.3389/fpls.2018.00264)
Supplement: Table S1 — Rg1 concentrations in soil in which Panax notoginseng was cultivated for 1–3 years. [file Table1.docx]

Table S1 Rg_1_ concentrations in soil in which *Panax notoginseng* was cultivated for 1-3 years

| Cultivated soil | Concentrations range (mg Kg^-1^) | Mean concentration  (mg Kg^-1^) |
| --- | --- | --- |
| One- year of consecutively cultivated bulk soil | 0.21-1.32 | 0.70 |
| Two- years of consecutively cultivated bulk soil | 0.83-1.54 | 1.09 |
| Three- years of consecutively cultivated bulk soil | 1.03-2.01 | 1.41 |
