# Supplementary material for: Panax notoginseng Root Cell Death Caused by the Autotoxic Ginsenoside Rg1 Is Due to Over-Accumulation of ROS, as Revealed by Transcriptomic and Cellular Approaches
Source: Front Plant Sci. 2018 Feb 28;9:264. doi: 10.3389/fpls.2018.00264 (PMC5836058; doi:10.3389/fpls.2018.00264)
Supplement: Table S2 — Summary of Illumina paired-end sequencing and assembly of root genes in Panax notoginseng. [file Table2.docx]

Table S2 Summary of Illumina pair-end sequencing and assembly of root genes in *Panax notoginseng*

| De novo assembly of reads | Total reads | 391600490 |
| --- | --- | --- |
|  | Total Nucleotides (nt) | 48950061250 |
|  | Q20 percentage | 94.39% |
|  | N percentage | 0.00% |
|  | GC percentage | 45.92% |
| Assembly of genes | genes number | 100125 |
|  | GC percent | 42.21 % |
|  | N50 | 945 |
|  | Max length | 12090 |
|  | Mini length | 201 |
|  | Average length | 631.82 |
|  | Total assembled bases | 63261077 |
