# Supplementary material for: Panax notoginseng Root Cell Death Caused by the Autotoxic Ginsenoside Rg1 Is Due to Over-Accumulation of ROS, as Revealed by Transcriptomic and Cellular Approaches
Source: Front Plant Sci. 2018 Feb 28;9:264. doi: 10.3389/fpls.2018.00264 (PMC5836058; doi:10.3389/fpls.2018.00264)
Supplement: Table S4 — Pathway enrichment analysis of DEGs in different treatments. [file Table4.DOCX]

Table S4 Pathway enrichment analysis of DEGs in different treatments

| Time | No. | Pathway | DEGs with pathway annotation | DEGs up regulated | DEGs down regulated | All genes with pathway annotation | P value | Q value | Pathway ID |
| --- | --- | --- | --- | --- | --- | --- | --- | --- | --- |
| 12h | 1 | Oxidative phosphorylation | 5 (27.78%) | 0 | 5 | 1071 (4.53%) | 0.001 | 0.009 | ko00190 |
|  | 2 | Ribosome | 8 (44.44%) | 0 | 8 | 4369 (18.48%) | 0.010 | 0.045 | ko03010 |
|  | 3 | Photosynthesis-antenna proteins | 1 (5.56%) | 1 | 0 | 38 (0.16%) | 0.029 | 0.086 | ko00196 |
|  | 4 | Glycerophospholipid metabolism | 1 (5.56%) | 0 | 1 | 163 (0.69%) | 0.117 | 0.260 | ko00564 |
| 24h | 1 | Ribosome | 230 (34.23%) | 173 | 57 | 4369 (18.48%) | 4.732e^-23^ | 3.833e^-21^ | ko03010 |
|  | 2 | Photosynthesis | 23 (3.42%) | 23 | 0 | 211 (0.89%) | 4.000e^-08^ | 1.620e^-06^ | ko00195 |
|  | 3 | Photosynthesis-antenna proteins | 10 (1.49%) | 10 | 0 | 38 (0.16%) | 7.402e^-08^ | 1.998e^-06^ | ko00196 |
|  | 4 | Linoleic acid metabolism | 4 (0.60%) | 4 | 0 | 34 (0.14%) | 0.015 | 3.088e^-01^ | ko00591 |
|  | 5 | RNA transport | 30 (4.46%) | 28 | 2 | 728 (3.08%) | 0.028 | 4.505e^-01^ | ko03013 |
| 48h | 1 | Oxidative phosphorylation | 5 (29.41%) | 0 | 5 | 1071 (4.53%) | 0.001 | 0.007 | ko00190 |
